# National Study of Firearm Presence and Storage Practices in Homes of Rural Adolescents

**DOI:** 10.5811/westjem.35252

**Published:** 2025-03-15

**Authors:** Benjamin Linden, Megan Sinik, Kristel Wetjen, Pam Hoogerwerf, Junlin Liao, Charles Jennissen

**Affiliations:** * University of Iowa, Roy J. and Lucille A. Carver College of Medicine, Iowa City, Iowa; † University of Minnesota Medical School, Department of Surgery, Minneapolis, Minnesota; ‡ Harvard Medical School, Beth Israel Deaconess Medical Center, Department of Internal Medicine, Boston, Massachusetts; § University of Iowa, University of Iowa Health Care, Department of Surgery, Iowa City, Iowa; ¶ University of Iowa, Roy J. and Lucille A. Carver College of Medicine, Stead Family Department of Pediatrics, Iowa City, Iowa; || University of Iowa Health Care Stead Family Children’s Hospital, Injury Prevention and Community Outreach, Iowa City, Iowa; # University of Iowa, Roy J. and Lucille A. Carver College of Medicine, Department of Emergency Medicine, Iowa City, Iowa

## Abstract

**Introduction:**

Firearm-related unintentional and suicide death rates in adolescents are higher in rural areas. In 2020, the overall rural firearm death rate was 28% higher than the urban rate. Firearm access significantly increases the risk. The study objective was to evaluate firearm exposure and storage practices in the homes of rural adolescents.

**Methods:**

We conducted a cross-sectional, anonymous survey of attendees at the 2021 National FFA (formerly Future Farmers of America) Convention & Exposition. Descriptive, bivariate, and multivariable logistic regression analyses were performed.

**Results:**

A total of 3,296 adolescents 13–18 years of age participated in our survey. Overall, 87% of respondents reported having rifles/shotguns, 71% had handguns, and 69% had both rifles/shotguns and handguns in their homes. The odds of those living on farms having rifles/shotguns and handguns were 7.5 and 2 times higher, respectively, as compared to those from towns. Rifles/shotguns and handguns were stored unlocked and/or loaded at least some of the time in 63% and 64% of homes, respectively. Respondents from farms had 1.5 and 1.7 times greater odds of having rifles/shotguns and handguns stored unlocked and loaded, respectively, as compared to those from town. The South, West and Midwest had odds that were 5.9, 3.2, and 2.8 times higher for rifles/shotguns and 8.1, 5.2, and 4.3 times greater for handguns to be stored loaded and unlocked, respectively, as compared to the Northeast. Only 43% of respondents reported ammunition being locked and stored separately from firearms.

**Conclusion:**

Most rural adolescents surveyed lived in homes with firearms, and a large proportion of those firearms were not stored safely. Firearm presence and storage differed by region and home setting. Unsafe storage practices could be contributing to the higher unintentional and suicide death rates seen in rural areas.

## INTRODUCTION

Firearm-related injuries are the leading cause of death in the United States for children and adolescents.^1^ There were 4,357 firearm-related deaths in this age group in 2020, which was a 30% increase from the previous year and more than double the rate of increase for other ages.^2^,^3^ Increases were seen for all types of firearm-related deaths—suicides, homicides, unintentional, and those undetermined.^2^,^3^ The US stands alone among developed countries for firearms being the leading cause of youth fatalities.^3^

Studies have shown that the presence of firearms in the home is associated with increased risk of firearm-related unintentional, homicide, and suicide deaths.^4^–^7^ Over half of all youth unintentional firearm deaths occur in the victim’s home, and the firearms used were stored loaded and unlocked in over 70% of the cases with known storage information.^8^ In addition, approximately 85% of firearm-related homicides in children ≤12 years of age have occurred in the home.^9^

Rural households are more likely to have firearms as compared to urban areas.^10^–^12^ Youth firearm-related suicide and unintentional death rates are also significantly higher in rural communities as compared to urban areas.^5^,^13^–^16^ In 2020, the rural firearm death rate was 28% higher than the urban rate, largely driven by rural firearm suicide deaths including youth.^17^ In fact, over the past three decades, over half of all adolescent suicides have been committed with firearms.^18^–^20^ In addition, the hospitalization rate for pediatric firearm-related injuries is higher for rural than for urban youth with unintentional injuries being the leading cause.^5^ Furthermore, the unintentional firearm death rate for rural teenagers aged 14–17 years is double the rate for urban adolescents.^15^

The American Academy of Pediatrics (AAP) guidelines for firearm-related anticipatory guidance states that firearms should be unloaded and locked, with ammunition locked separately from the firearm.^21^ It has been estimated that locking all firearms could reduce youth firearm deaths by up to 32%.^22^ Understanding demographic differences in firearm ownership and storage practices is important when developing strategies for effective anticipatory guidance and firearm-injury prevention programs. Our objective in this study was to determine firearm exposure and storage practices in the homes of rural adolescents, and to identify factors associated with unsafe firearm storage. This expands upon our earlier study conducted among rural adolescents in Iowa.^12^

## METHODS

### Study Population

A cross-sectional survey was conducted of a convenience sample of adolescents attending the 2021 National FFA (formerly Future Farmers of America) Convention & Exposition in Indianapolis, Indiana. The FFA has nearly 950,000 members in over 9,000 chapters in all 50 states, Puerto Rico, and the US Virgin Islands. Members are typically in the 5^th^-12^th^ grade. Around 60,000 students, teachers, administrators, former FFA members, exhibitors, and guests attended the conference. Conference attendees completed an anonymous survey at the study institution’s injury prevention booth. Team members administered the surveys to respondents who were instructed to complete them independently. Survey data was entered into Qualtrics (Qualtrics International, Inc, Provo, UT). As an incentive for completing the survey, participants were provided a modest prize (eg, lip balm, trucker cap) as determined by chance via a spinning wheel.

**Population Health Research Capsule**

What do we already know about this issue?

*Rural households are more likely to have firearms in the home compared to urban. Youth firearm suicide and unintentional death rates are higher in rural areas.*

What was the research question?

*What factors are associated with unsafe firearm storage in the homes of rural adolescents?*

What was the major finding of the study?

*Nearly 90% of rural adolescents surveyed reported having at least one firearm in their home and over 60% endorsed some form of unsafe storage.*

How does this improve population health?

*Most rural youth live in households with unsafe firearm storage. Targeted efforts to increase safe storage may prevent youth unintentional injury and suicide.*

### Survey

The survey was developed by the study institution’s Firearm Safety Task Force. Demographic data collected included age (years), gender (male, female, other, choose not to respond), residence (on a farm, in the country but not on a farm, in town), race/ethnicity (White, Black, Hispanic Latinx, Asian, Native Hawaiian/Pacific Islander, Native American/Alaska Native, mixed, other) and the state of residence. In the survey, the term “firearm” was defined as a weapon “from which a bullet or other projectile is fired by gunpowder,” and did not include BB guns, pellet guns, or dart guns. The term “home” included “the place you sleep and all other buildings your family owns on the same property.” A firearm was considered “loaded” if there was ammunition in the firearm including the magazine, tube, chamber or other. A firearm was considered “unlocked” if it was “not locked in a storage place or not stored with a trigger lock or cable.”

The survey asked whether there were any rifles/shotguns or handguns in the home. Answer choices included “yes” and “not that I know of.” For respondents who answered “yes,” they were then asked if the firearms were stored “loaded,” “unlocked,” or “both loaded and unlocked.” Respondents could select “yes, always,” “yes, sometimes,” “no, never,” or “not sure” for each of the storage questions. The survey asked how the ammunition was stored with answer options including “locked with firearms,” “locked separately,” “not locked,” and “not sure.” For the purposes of analysis, safe storage was defined as the firearm being stored unloaded and locked.

### Data Analysis

Completed surveys were provided by the Firearm Safety Task Force to the research team for analysis. The study institution’s institutional review board deemed the research exempt as the analysis was performed on an anonymously collected existing dataset. Aggregate survey results were exported as an Excel spreadsheet (Microsoft Corp, Redmond, WA) and imported into Stata 15.1 (StataCorp, College Station, TX). Analysis was limited to those ages 13–18 years. We performed descriptive (frequencies), bivariate (chi square, Fisher exact test), and multivariable logistic regression analyses. All *P*-values were two-tailed, and a value <0.05 was considered statistically significant. Missing data were not included in the analyses.

## RESULTS

### Subject Demographics

A total of 3,296 adolescents completed the survey. The proportion of males and females was nearly equivalent, and 66% were 16–18 years of age (Table 1). Nearly three-quarters lived outside of a town. Over 90% were non-Hispanic (NH) White. Respondents were from Puerto Rico and every US state except Maine, Massachusetts, New Hampshire, and Vermont. About two-thirds of participants lived in the Midwest US Census Region, one-fifth lived in the South, and <10% each lived in the West and Northeast.

### Firearms in the Home

The vast majority (87%) reported having at least one rifle/shotgun, and 71% reported having at least one handgun in their home. Approximately 70% of respondents reported the presence of both a rifle/shotgun and a handgun in the home. A small percentage reported having handguns only, and 18% reported having rifles/shotguns only. Overall, 89% reported having at least one firearm (rifle/shotgun and/or handgun) in the home.

### Demographic Comparisons of Rifle/Shotgun Presence in the Home

Males, those living on a farm or in the country/not a farm, and NH Whites all had higher proportions with rifles/shotguns in their home relative to their peers (Table 2). The odds of males reporting rifles/shotguns in their homes was twice as high as females. Individuals living on a farm and living in the country/not a farm had odds that were 7.6 and 3.5 times greater than those living in town, respectively. Individuals identifying as other races than NH White had odds that were 70% lower than respondents who identified as NH White. The odds of rifles/shotguns in the home were more than twice as high for those residing in the Midwest, South, or West US Census regions as compared to those in the Northeast.

### Demographic Comparisons of Handgun Presence in the Home

For the presence of handguns in the home, males, those living on a farm or in the country/not a farm, and NH Whites all had higher proportions relative to their peers (Table 2). Males had odds about 1.5 times greater than females of having a handgun in the home. Those living on a farm or in the country/not a farm both had odds that were twice that of those living in town. Respondents of other races than NH White had odds about 40% lower than NH Whites. The odds of handguns in the home were 1.6, 3.0, and 3.6 times higher for respondents from the Midwest, West, and South US Census regions, respectively, as compared to the Northeast.

### Firearm Storage Practices in the Home

Over one-third of respondents reported that rifles/shotguns were stored loaded at least some of the time, and half reported they were sometimes or always stored unlocked (Table 3). Among all respondents aware of rifle/shotgun storage practices in their homes, over one-fifth reported they were stored locked and unloaded at least some of the time. Overall, over three-fifths of respondents reported unsafe rifle/shotgun storage in the home. For those reporting handguns in the home, nearly half reported their handguns being stored loaded at least some of the time (Table 3). About half also reported handguns being stored unlocked at least sometimes. Moreover, just under one-third of respondents reported handguns being stored both loaded and unlocked at least some of the time. Overall, nearly two-thirds of participants reported unsafe handgun storage in the home.

### Demographic Comparisons of Rifle/Shotgun Storage in the Home

Males had higher proportions of reporting rifles/shotguns being stored unlocked in the home (Table 4). The odds of males reporting rifles/shotguns being stored unlocked and stored loaded/unlocked were 1.7 and 1.3 times greater, respectively, than females. Older teens (16–18 years) had higher proportions and greater odds (1.3 times) of reporting rifles/shotguns as being stored unlocked as compared to younger teens. Those living on a farm had higher proportions reporting unsafe rifle/shotgun storage than those who lived elsewhere. The odds of having rifles/shotguns stored loaded, unlocked, and loaded and unlocked were 1.4, 1.7 and 1.5 times greater, respectively, for those living on a farm vs those from towns.

There were differences in rifle/shotgun storage by US Census Region with respondents from the South having higher proportions and those from the Northeast having lower proportions reporting unsafe storage. Moreover, youth from the South had 3.4 and 1.9 times higher odds of reporting rifles/shotguns being stored loaded and being stored unlocked, respectively, as compared to those from the Northeast. Similarly, respondents from the West had 1.8 times higher odds of reporting rifles/shotguns being stored unlocked relative to those from the Northeast. Finally, the odds of youth reporting having firearms being stored both loaded and unlocked for the Midwest, West and South were 2.8, 3.2 and 5.9 times greater, respectively, as compared to respondents from the Northeast.

### Demographic Comparisons of Handgun Storage in the Home

Many similarities were seen in the demographics of reporting unsafe rifle/shotgun and unsafe handgun storage (Table 5). There were higher proportions of males reporting unsafe handgun storage with the odds of males reporting handguns being stored loaded, unlocked, and loaded/unlocked being 1.2, 1.5 and 1.7 times greater, respectively, than females. Older teens had higher proportions reporting unsafe handgun storage in the home, with the odds of older teens reporting handguns being stored unlocked and loaded/unlocked being 1.5 and 1.4 times higher, respectively, than younger teens. The odds of having handguns stored unlocked were 1.8 times greater for those from farms and 1.4 times higher for those from the country/not a farm as compared to those from towns. Similarly, those from farms and from the country/not a farm both had 1.7 times greater odds of handguns in the home being stored loaded and unlocked vs respondents from towns.

Youth from the South US Census Region had the highest proportions reporting unsafe handgun storage, whereas those from the Northeast had the lowest. Moreover, the odds of youth reporting handguns being stored loaded were 2.6, 2.6 and 5.2 times greater for the Midwest, West and South, respectively, as compared to the Northeast. Similarly, youth from the West and South had 1.9 and 2.3 times higher odds of reporting handguns being stored unlocked, respectively, relative to the Northeast. In addition, the odds of participants reporting handguns being stored loaded and unlocked were 4.3, 5.2 and 8.1 times greater, respectively, for the Midwest, West and South as compared to the Northeast.

### Ammunition Storage Practices

Only 43% of respondents with known rifles/shotguns in the home reported that the ammunition was always locked and stored separately from the firearms, and 22% of respondents reported ammunition was not locked at all. Males and respondents aged 16–18 years both had 1.5 times greater odds of reporting ammunition being stored unlocked as compared to females and younger teens (data not shown in a table). There were no observed differences in ammunition storage practices by subject’s race/ethnicity, residence, or US Census Region.

The storage practices for handgun ammunition were similar to those of rifles/shotguns in that 41% reported ammunition being locked and stored separately from the handguns, and 22% reporting handgun ammunition as not being locked. Males and those aged 16–18 years had higher proportions with unlocked handgun ammunition in the home with odds 1.4 and 1.7 times greater, respectively, of reporting unlocked ammunition than females or younger teens. The odds of respondents living on a farm reporting unlocked handgun ammunition was 1.5 times higher as compared to those living in town. There were no observed differences by race/ethnicity or across US Census regions.

## DISCUSSION

A large majority of survey respondents reported having at least one firearm in their home. Firearm presence in the home was higher in the South, West and Midwest compared to the Northeast for both rifles/shotguns and handguns. Over one-fifth reported that rifles/shotguns were stored both loaded and unlocked at least some of the time, and nearly one-third reported having handguns stored loaded and unlocked at least some of the time. More than three-fifths of those with firearms in the home reported having unsafe storage at least some of the time.

The population of this study is best described as rural adolescents. Several studies have shown that rural settings have higher rates of unintentional and suicide firearm deaths including among youth.^13^,^14^,^23^,^24^ Unsafe storage has been associated with an increased risk of youth firearm suicide.^25^–^27^ It is estimated that ~34% of all children in the US live in homes with firearms, and 13% of these homes have firearms that are stored unsafely.^28^ Our study respondents had higher proportions of firearm ownership and unsafe storage as compared to the general population. This may contribute to the higher rates of firearm-related injury and suicide seen in rural areas of the US.^29^

Respondents from the South had higher proportions and greater odds of both rifles/shotguns and handguns being stored loaded and unlocked relative to other regions. This was also observed in the 2019 National Firearm Survey where US adults in the South had twice the odds for storing firearms loaded and unlocked as compared to the Northeast, an odds ratio comparatively higher than that of the Midwest and West as well.^30^ Conversely, rural youth from the Northeast had lower proportions whose families owned firearms and stored them unsafely. This difference in firearm ownership is consistent with a 2017 Pew Research Center survey that found 16% of adults living in the Northeast reported owning a gun, half the ownership rate of adults in the South (36%), Midwest (32%) and West (31%).^11^ Six of the 10 states with the highest youth firearm death rates are in the South US Census Region, and the four states with the lowest rates are in the Northeast.^17^ This regional pattern correlates with our study’s firearm ownership and storage results. The overlap of higher firearm deaths and unsafe storage practices raises concern for a possible causal relationship; unsafe storage increases access to firearms and risk of possible injury.

Our study shows a high prevalence of firearms that are unsafely stored in homes of rural adolescents. A proven strategy for reducing the risk of firearm-related death is the passage of state child access prevention (CAP) laws that impose criminal liability on adults who fail to prevent children from unauthorized access to firearms. State CAP laws are associated with reductions in firearm mortality including suicide rates among youth.^31^,^32^ Moreover, states with strong CAP laws have lower rates of firearm-related suicide and unintentional injuries in adolescents as compared to states without CAP laws.^18^,^33^–^35^ Approximately 75% of adults and youth support such measures.^12^,^36^,^37^ None of the 10 states with the highest youth firearm death rate have implemented CAP laws whereas 9 of the 10 states with the lowest youth firearm death rate have some form of CAP law in place.^38^

Anticipatory guidance has been shown to be effective in increasing parents’ awareness of risks to their child’s health and to promote safer practices.^39^–^41^ Clinicians counseling patients can effectively promote safe storage practices, especially when offering a free, safe firearm storage device (ie, trigger lock).^42^ The AAP recommends that pediatricians routinely discuss firearm safety with patients and their families in the context of injury prevention, including encouraging parents to ask about firearms and their storage in homes their children visit.^43^

Future studies should include focus group discussions to better understand rural families’ attitudes regarding firearm storage, what practices households would be willing to change to increase safety, and what messaging they believe would be effective in educating rural families to improve storage practices and help decrease rural firearm-related injuries. Ultimately, a multifaceted approach that includes continued safety education, clinician counseling, and policy will be essential to reduce firearm-related injury and death among our rural youth.

## LIMITATIONS

The study used a convenience sampling of US adolescents at the National FFA Leadership Conference who were primarily from rural areas. Thus, the study does not represent urban areas of the country. In 2021, according to the US Department of Agriculture, the percentage of farms by US Census Region was as follows: Midwest 36%; South 42%; West 16%; and Northeast 6%.^44^ Nearly two-thirds of our respondents were from the Midwest suggesting an oversampling from this region, likely explained by the conference being held within this region. In addition, over 90% of respondents were NH White, which is a higher proportion than that determined by the 2020 US Census for rural residents (76%).^45^ Thus, our findings may not be generalizable to all states and to non-White populations. Responses to the survey were self-reported and could be affected by recall bias and social desirability. However, surveys were completed independently and collected anonymously, which should have helped decrease the social desirability bias.

## CONCLUSION

A significant majority of rural youth in our study lived in households with firearms. Most reported some form of unsafe storage. There were differences regarding the presence and storage of firearms by demographics, especially the US Census Region and home setting. Unsafe storage practices could be contributing to the higher unintentional and suicide death rates seen in rural areas. Widespread efforts are needed to educate and counsel rural families about the importance of proper firearm storage and must consider the unique cultural and social aspects of rural communities.

## Figures and Tables

**Table 1:** Demographic and firearm-related variables of adolescent survey respondents.

	n (column %)^a^
Group N	3,296
Sex	
Male	1,623 (50)
Female	1,639 (50)
Other	16 (<1)
Age	
13 years	60 (2)
14 years	327 (10)
15 years	710 (22)
16 years	890 (27)
17 years	947 (29)
18 years	353 (11)
Residence	
Farm	1,495 (45)
Country/not a farm	1,116 (34)
Town	679 (21)
Race/Ethnicity	
Non-Hispanic White	3,025 (92)
Other races/ethnicities	261 (8)
US Census regions	
South	692 (21)
West	294 (9)
Midwest	2,173 (67)
Northeast	11 (3)
Rifle/shotgun in home	
Yes	2,856 (87)
Not that I know of	436 (13)
Handgun in home	
Yes	2,342 (71)
Not that I know of	946 (29)
Combined firearms in home	
Both rifle and handgun	2,275 (69)
Rifle/shotgun only	581 (18)
Handgun only	67 (2)
None that I know of	369 (11)

a The sum of n may not equal the total Group N due to missing values.

**Table 2:** Bivariate and multivariate logistic regression analyses regarding the presence of rifles/shotguns and handguns in the homes of adolescent survey respondents.

	Rifle/shotgun in the home			Logistic regression analysis^a^	
	
	Yes n (row %)^c^	No^b^ n (row %)^c^	*P*-value	Odds ratio	Confidence interval
	
Group N	2,856 (87)	436 (13)			
Sex					
Male	1,483 (92)	138 (9)	<0.001	2.08	1.65–2.62
Female	1,350 (83)	287 (18)		1.0 (ref)	
Age					
16–18 years	1,887 (86)	301 (14)	0.20	0.91	0.71–1.15
13–15 years	962 (88)	133 (12)		1.0 (ref)	
Residence					
Farm	1,407 (94)	86 (6)	<0.001	7.56	5.69–10.05
Country/not a farm	988 (89)	128 (12)		3.64	2.81–4.70
Town	456 (67)	221 (33)		1.0 (ref)	
Race/ethnicity					
Non-Hispanic White	2,671 (88)	352 (12)	<0.001	1.0 (ref)	
Other races/ethnicities	178 (68)	83 (32)		0.30	0.21–0.41
U.S. Census Region					
South	600 (87)	92 (13)	0.01	2.51	1.47–4.29
West	255 (87)	39 (13)		2.76	1.50–5.07
Midwest	1,900 (88)	271 (12)		2.34	1.43–3.84
Northeast	85 (77)	26 (23)		1.0 (ref)	

	Handgun in the home			Logistic Regression Analysis^a^	
	
	Yes n (row %)^c^	No^b^ n (row %)^c^	*P*-value	Odds ratio	Confidence Interval
	
Group N	2,342 (71)	946 (29)			
Sex					
Male	1,231 (76)	387 (24)	<0.001	1.51	1.29–1.77
Female	1,092 (67)	544 (33)		1.0 (ref)	
Age					
16–18 years	1,539 (70)	647 (30)	0.13	0.87	0.73–1.03
13–15 years	797 (73)	296 (27)		1.0 (ref)	
Residence					
Farm	1,114 (75)	377 (25)	<0.001	2.02	1.65–2.46
Country/not a farm	829 (74)	285 (26)		1.97	1.60–2.44
Town	395 (58)	282 (42)		1.0 (ref)	
Race/ethnicity					
Non-Hispanic White	2,173 (72)	846 (28)	0.001	1.0 (ref)	
Other races/ethnicities	162 (62)	99 (38)		0.61	0.46–0.82
US Census Region					
South	564 (82)	128 (18)	p<0.001	3.55	2.30–5.48
West	229 (78)	64 (22)		3.00	1.85–4.86
Midwest	1,474 (68)	694 (32)		1.59	1.07–2.37
Northeast	63 (57)	48 (43)		1.0 (ref)	

a This analysis controlled for all other variables in the table.

b The actual response was “Not that I know of” as homes may have had firearms, but the adolescent respondent may not have known that they were present.

c The sum of n for a variable may not equal the total Group N due to missing values.

**Table 3:** Storage of firearms and of handguns in the homes of adolescent survey respondents.

	Rifles/shotguns n (column %)^a^	Handguns n (column %)^b^
Stored loaded		
No	1,680 (64)	1,131 (52)
Yes, always	332 (13)	469 (21)
Yes, sometimes	632 (24)	578 (27)
Stored unlocked		
No	1,357 (50)	1,126 (51)
Yes, always	486 (18)	401 (18)
Yes, sometimes	859 (32)	674 (31)
Stored loaded and unlocked		
No	2,055 (78)	1,466 (68)
Yes, always	164 (6)	246 (11)
Yes, sometimes	426 (16)	452 (21)
Overall storage		
Safe storage^c^	950 (37)	764 (36)
Unsafe storage^d^	1,603 (63)	1,348 (64)

a Does not include those who had no rifles/shotguns in the home or were unsure of storage.

b Does not include those who had no handguns in the home or were unsure of storage.

c Firearms always stored unloaded and locked.

d Firearms stored at least sometimes loaded and/or unlocked.

**Table 4:** Bivariate and multivariable logistic regression analyses regarding the storage of rifles/shotguns in the homes of adolescent survey respondents.^a^

	Rifle storage			Logistic regression analysis^b^	
	
	Yes^c^ n (row %)^d^	No n (row %)^d^	P-value	Odds ratio	Confidence interval
Stored loaded					
Sex					
Male	519 (36)	907 (64)	0.90	0.96	0.81–1.13
Female	439 (37)	759 (63)		1.0 (ref)	
Age					
16–18 years	647 (36)	1129 (64)	0.93	0.97	0.81–1.15
13–15 years	316 (37)	547 (63)		1.0 (ref)	
Residence					
Farm	505 (38)	832 (62)	0.06	1.35	1.06–1.72
Country/not a farm	333 (37)	567 (63)		1.26	0.98–1.63
Town	126 (31)	277 (69)		1.0 (ref)	
Race/ethnicity					
Non-Hispanic White	900 (36)	1581 (64)	0.30	1.0 (ref)	
Other races/ethnicities	63 (40)	93 (60)		1.18	0.83–1.67
US Census Region					
South	286 (52)	268 (48)	<0.001	3.43	1.96–5.99
West	73 (3)	162 (69)		1.49	0.82–2.72
Midwest	580 (33)	1183 (67)		1.59	0.93–2.74
Northeast	19 (24)	59 (76)		1.0 (ref)	
Stored unlocked					
Sex					
Male	807 (56)	641 (44)	<0.001	1.67	1.43–1.95
Female	527 (43)	706 (57)		1.0 (ref)	
Age					
16–18 years	932 (52)	861 (48)	0.002	1.31	1.11–1.55
13–15 years	413 (46)	489 (54)		1.0 (ref)	
Residence					
Farm	733 (54)	618 (46)	<0.001	1.73	1.38–2.17
Country/not a farm	436 (47)	487 (53)		1.31	1.03–1.66
Town	175 (41)	248 (59)		1.0 (ref)	
Race/Ethnicity					
Non-Hispanic White	1262 (50)	1271 (50)	0.91	1.0 (ref)	
Other races/ethnicities	80 (49)	82 (51)		0.94	0.67–1.31
US Census Region					
South	310 (55)	258 (45)	0.009	1.85	1.13–3.04
West	133 (54)	113 (46)		1.77	1.04–3.02
Midwest	861 (48)	931 (52)		1.42	0.88–2.28
Northeast	33 (41)	47 (59)		1.0 (ref)	
Stored loaded and unlocked					
Sex					
Male	348 (25)	1,069 (75)	0.004	1.29	1.07–1.56
Female	239 (20)	966 (80)		1.0 (ref)	
Age					
16–18 years	415 (24)	1,354 (76)	0.05	1.20	0.98–1.48
13–15 years	175 (20)	695 (80)		1.0 (ref)	
Residence					
Farm	327 (25)	1,000 (75)	0.01	1.45	1.09–1.93
Country/not a farm	187 (21)	714 (79)		1.14	0.85–1.55
Town	76 (19)	336 (82)		1.0 (ref)	
Race/Ethnicity					
Non-Hispanic White	546 (22)	1934 (78)	0.13	1.0 (ref)	
Other races/ethnicities	43 (27)	115 (72)		1.38	0.94–2.02
US Census Region					
South	187 (34)	369 (66)	<0.001	5.90	2.51–13.86
West	51 (21)	187 (79)		3.16	1.30–7.72
Midwest	342 (19)	1415 (81)		2.80	1.20–6.52
Northeast	6 (8)	73 (92)		1.0 (ref)	

a Those who answered “unsure” regarding firearm storage were not included in that analysis.

b This analysis controlled for all other variables in the table.

c Includes those who answered “Yes, always” and “Yes, sometimes.”

d The sum of n for a variable may not equal the total Group N due to missing values.

**Table 5:** Bivariate and multivariable logistic regression analyses regarding the storage of handguns in the homes of adolescent survey respondents.^a^

	Handgun storage			Logistic regression analysis^b^	
	
	Yes^c^ n (row %)^d^	No n (row %)^d^	P-value	Odds ratio	Confidence interval
Stored loaded					
Sex					
Male	602 (51)	588 (49)	0.01	1.22	1.03–1.46
Female	438 (45)	533 (55)		1.0 (ref)	
Age					
16–18 years	702 (49)	747 (52)	0.62	1.04	0.87–1.25
13–15 years	343 (47)	382 (53)		1.0 (ref)	
Residence					
Farm	496 (47)	556 (53)	0.15	1.07	0.83–1.37
Country/not a farm	392 (51)	379 (49)		1.24	0.95–1.60
Town	159 (45)	192 (55)		1.0 (ref)	
Race/ethnicity					
Non-Hispanic White	978 (48)	1048 (52)	0.63	1.0 (ref)	
Other races/ethnicities	67 (46)	78 (54)		0.90	0.63–1.28
US Census Region					
South	327 (62)	201 (38)	<0.001	5.15	2.75–9.63
West	94 (44)	119 (56)		2.61	1.35–5.05
Midwest	603 (44)	761 (56)		2.58	1.40–4.75
Northeast	15 (25)	46 (75)		1.0 (ref)	
Stored unlocked					
Sex					
Male	637 (53)	558 (47)	<0.001	1.45	1.22–1.73
Female	429 (43)	560 (57)		1.0 (ref)	
Age					
16–18 years	754 (52)	703 (48)	<0.001	1.46	1.21–1.75
13–15 years	318 (43)	421 (57)		1.0 (ref)	
Residence					
Farm	571 (53)	501 (47)	<0.001	1.79	1.40–2.30
Country/not a farm	362 (47)	400 (53)		1.40	1.08–1.82
Town	142 (39)	221 (61)		1.0 (ref)	
Race/ethnicity					
Non-Hispanic White	1000 (49)	1042 (51)	0.82	1.0 (ref)	
Other races/ethnicities	73 (48)	79 (52)		0.93	0.66–1.32
US Census Region					
South	305 (58)	223 (42)	<0.001	2.28	1.31–3.96
West	119 (54)	102 (46)		1.88	1.04–3.39
Midwest	620 (45)	757 (55)		1.35	0.79–2.31
Northeast	24 (38)	39 (62)		1.0 (ref)	
Stored loaded and unlocked					
Sex					
Male	445 (37)	741 (63)	<0.001	1.69	1.40–2.05
Female	251 (26)	711 (74)		1.0 (ref)	
Age					
16–18 years	489 (34)	945 (66)	0.01	1.35	1.11–1.65
13–15 years	208 (29)	517 (71)		1.0 (ref)	
Residence					
Farm	358 (34)	692 (66)	<0.001	1.65	1.25–2.19
Country/not a farm	256 (34)	496 (66)		1.68	1.25–2.25
Town	84 (23)	274 (77)		1.0 (ref)	
Race/Ethnicity					
Non-Hispanic White	649 (32)	1364 (68)	0.70	1.0 (ref)	
Other races/ethnicities	49 (34)	96 (66)		1.07	0.73–1.56
US Census Region					
South	227 (43)	299 (57)	<0.001	8.05	3.15–20.56
West	71 (33)	145 (67)		5.23	1.99–13.73
Midwest	388 (29)	962 (71)		4.32	1.71–10.95
Northeast	5 (8)	55 (92)		1.0 (ref)	

a Those who answered “unsure” regarding firearm storage were not included in that analysis.

b This analysis controlled for all other variables in the table.

c Includes those who answered “Yes, always” and “Yes, sometimes.”

d The sum of n for a variable may not equal the total Group N due to missing values.
